# Chitin and Chitosan Derivatives as Biomaterial Resources for Biological and Biomedical Applications

**DOI:** 10.3390/molecules25245961

**Published:** 2020-12-16

**Authors:** Saravut Satitsri, Chatchai Muanprasat

**Affiliations:** Chakri Naruebodindra Medical Institute, Faculty of Medicine Ramathibodi Hospital, Mahidol University, Bang Phli, Samut Prakarn 10540, Thailand; saravut.sat@mahidol.ac.th

**Keywords:** chitin, chitosan, chitosan oligosaccharide

## Abstract

Chitin is a long-chain polymer of *N*-acetyl-glucosamine, which is regularly found in the exoskeleton of arthropods including insects, shellfish and the cell wall of fungi. It has been known that chitin can be used for biological and biomedical applications, especially as a biomaterial for tissue repairing, encapsulating drug for drug delivery. However, chitin has been postulated as an inducer of proinflammatory cytokines and certain diseases including asthma. Likewise, chitosan, a long-chain polymer of *N*-acetyl-glucosamine and d-glucosamine derived from chitin deacetylation, and chitosan oligosaccharide, a short chain polymer, have been known for their potential therapeutic effects, including anti-inflammatory, antioxidant, antidiarrheal, and anti-Alzheimer effects. This review summarizes potential utilization and limitation of chitin, chitosan and chitosan oligosaccharide in a variety of diseases. Furthermore, future direction of research and development of chitin, chitosan, and chitosan oligosaccharide for biomedical applications is discussed.

## 1. Introduction

### 1.1. Chitin

Chitin is long-chain polymers of *N*-acetylglucosamine, presence of which has been experimentally confirmed in unicellular (diatoms, protists, fungi) as well as in multicellular (sponges, corals, mollusks, worms and arthropods) organisms [1]. Chitin has been used as supplementary for nutraceutical food, pharmaceutical products as well as 3D scaffolds for biomedicine [2,3,4] and technological applications [5,6,7,8]. Chitin contains thermostability which can be synthesized in the high temperature process [9]. Moreover, chitin has a high tolerance to high chemical concentration that generates the deposition of metals such as copper into chitin via electrochemical procedure at room temperature [10]. The linear polymer of chitin contains β-(1,4)-*N*-acetyl-d-glucosamines, which are linked by glycosidic bond. There are three isoforms of chitin including α-chitin, β-chitin and γ-chitin [11]. The different forms of chitin depend on the arrangement of side chain of backbone. For instance, the structure of α-chitin is parallel chain arrangement, whereas the structure of β-chitin is antiparallel chain arrangement. α-chitin is frequently found in nature including exoskeleton of arthropods. α-chitin has been frequently applied for tissue engineering [12,13]. In contrast to α-chitin, β-chitin is mostly found in squid pen. β-chitin has been also applied for biomaterial usage such as wound healing [14,15], preservative agents for methylene blue [16], and biological application such as an enhancer of saltiness perception [17]. γ-chitin has been gathered from cocoon of the moth (*Orgyia dubia*), and it is found that the structure of γ-chitin is similar to α-chitin rather than β-chitin, but the surface morphology of γ-chitin is consisted of microfibers, whereas α-chitin and β-chitin are composed of nanofibers [18]. 

### 1.2. Chitosan

Chitosan is derived from chitin by deacetylation. The major component of chitosan is the mixture between *N*-acetyl-d-glucosamine and β-(1,4)-linked-d-glucosamine. Chitosan has amounts of *N*-acetyl-d-glucosamine less than β-(1,4)-linked-d-glucosamine [19]. Chitosan is generally applied as biomaterials, especially for drug delivery system and use in combination with other substances for improving their therapeutic effects [20,21]. 

### 1.3. Chitosan Oligosaccharide

Chitosan oligosaccharide or chitooligosaccharide is oligomers of chitosan. Chitosan oligosaccharide has the degree of polymerization of <55 and molecular weight of <10 kDa [22]. Chitosan oligosaccharides have a variety of biomedical applications such as drug delivery system [20], functional food [23], as well as the drug against acne vulgaris [24]. 

In this review, we emphasize the potential application of chitin, chitosan and their oligosaccharides as therapeutics and useful biomaterials in a variety of human diseases. Furthermore, we discuss the limitation of chitin and chitosan oligosaccharide, which will be helpful in guiding future direction of research and development based on these molecules. 

## 2. Potential Applications of Chitin and Chitosan Oligosaccharides 

### 2.1. Neurological and Musculoskeletal Diseases

Chitin has been proposed as biomaterials for neural treatment. For instance, chitin was utilized with carbon nanotube as a scaffold for neural growth [25]. Chitin as a biological absorbable tube or catheter was effectively used as the bridge for sural nerve grafts in a rat sciatic nerve defect model [26,27]. Chitin hydrogel repaired cartilage injury by protecting chondrocytes from apoptosis and promoting immunomodulation of macrophage and chondrogenesis [28]. The combination of chitin as 2,2,6,6-Tetramethylpiperidine-1-oxyl (TEMPO)-oxidized sacchachitin nanofibers (TOSCNFs) and chitosan-activated platelet-rich plasma (cPRP) induced healing effect in corneal damage by promoting cell proliferation and cell migration in Statens Seruminstitut rabbit corneal (SIRC) epithelial cells [29]. Interestingly, chitin is not only proposed as potential therapy, but also claimed as a molecular marker for neurological diseases. In Alzheimer’s disease, chitin is elevated and accumulated within the brain and facilitates a scaffolding for amyloid-β deposition [30,31,32]. Fungal chitin was also detected in brain tissues from Alzheimer’s disease patients [33]. Moreover, chitin accumulation was found in multiple sclerosis patients [34]. Interestingly, both microglia and neurons produce *N*-acetylglucosamine polymerization, which causes neurotoxicity in Alzheimer’s disease [35]. Furthermore, chitin derived from demosponge *Aplysina aerophoba* was used as 3D scaffold for human bone marrow-derived stromal cells, and it promoted cell proliferation, cell bridging formation and metabolic activity with no toxicity [36]. Interestingly, chitin derived from demosponge *Ianthella basta* was applied as a cryopreservative agent that effectively retained adipogenic differentiation in human mesenchymal stromal cells [4].

In contrast to chitin, chitosan and chitosan oligosaccharide have been widely studied for the beneficial effect on neurological diseases, especially Alzheimer’s disease. A water-soluble form of chitosan inhibited the production of proinflammatory cytokines including TNFα and IL-6 as well as inducible nitric oxide synthase (iNOS) in human astrocytoma cell line CCF-STTG1 stimulated with IL-1β and Aβ fragments (25–35) [37]. It is known that acetylcholinesterase inhibition is the target for Alzheimer’s disease therapy. Chitosan oligosaccharide with 90% deacetylation and low molecular weight (1 to 5 kDa) inhibited the protein expression of acetylcholinesterase and Aβ fragment (25–35)-induced acetylcholinesterase activity in PC12 cell lines [38]. Furthermore, caffeic acid conjugated chitosan oligosaccharide effectively inhibited β-site amyloid precursor protein-cleaving enzyme activity, which was the rate limiting step of Aβ peptide formation in Alzheimer’s disease [39]. Interestingly, chitosan oligosaccharide at 500 µg/mL inhibited β-site amyloid precursor protein cleaving enzyme 1 (BACE1) activity and protein expression in HEK293 APPswe cells [40]. Chitosan oligosaccharide inhibited αβ aggregation, inhibited Aβ1–42 fibrils formation, and induced fibril destabilization in oligomeric Aβ-induced neurotoxicity and oxidative stress in rat hippocampal neurons [41]. In addition, 0.1% of chitosan oligosaccharide injected into the spatium intermusculare around the biceps femoris muscle inhibited scar formation and promoted regeneration of axons, as well as sensory and motor function in a mouse model of sciatic nerve injury [42]. Interestingly, ingestion of chitosan oligosaccharide (10 mg/kg/day) alleviated inflammatory signal including COX-2 expression in the synovium of an anterior cruciate ligament (ACL) transection-induced osteoarthritis rabbit model [43]. Ingestion of chitosan oligosaccharide at least 200 mg/kg/day recovered cognitive deficiency in Aβ1–42-induced learning and memory loss rats [44]. Chitosan oligosaccharide also protected hippocampal neuron from Aβ peptide [45]. Furthermore, low molecular weight chitosan activated mitogenic response to platelet-derived growth factor (PDGF) in vascular smooth muscle cells [46].

Chitosan has been applied as biomaterials for neural therapy. For instance, amphiphatic carboxymethyl-hexanoyl chitosan hydrogel increased cell viability and maintained stem-cell-like gene expression of induced pluripotent stem cells applied for corneal reconstruction [47]. Chitosan-polylactide fiber was utilized for nerve growth factor in PC12 cell lines [48]. Chitosan oligosaccharide with calcium silicate and gelatin was applied for implantation of cortical bone repair and bone fracture fixation [49]. Chitosan was coated into nanoparticles for delivering antiamyloid antibody as a drug for Alzheimer’s disease. It was found that chitosan coating improved aqueous dispersibility and stability of vehicle during lyophilization [50]. Chitosan in the form of chitosan beads effectively interfered with amyloid-β aggregation [51]. Chitosan was utilized as nanocapsules for delivering p38 inhibitor to the brain by nasal administration [52]. Chitosan with polyvinyl alcohol nanofibrous scaffold promoted skeletal muscle regeneration by increasing cell viability, cell adhesion, cell growth, and cell spread on the scaffold [53]. Chitosan with laminin and poly (lactic-co-glycolic acid) effectively repaired nerve injury by promoting nerve regeneration [54]. Chitosan combined to hyaluronate regenerated nerve function defect in parotidectomy rabbit model by promoting scar formation, increasing nerve fibers, thickening myelin sheath, and promoting nerve conduction velocity [55]. Furthermore, chitosan, as chitosan tubes or incorporated to mesenchymal stem cells or keratin, was also utilized for nerve repairing [56,57,58].

### 2.2. Cardiovascular and Hematological Diseases

Chitin has been incorporated with other substances for treatment of cardiovascular disease. For instance, chitin with glucan and polyphenols from pomegranate recovered endothelial dysfunction by reducing inflammatory marker in the liver and adipose tissues and promoting NO synthase in apolipoprotein E deficient mice (apoE−/−) with high fat diet [59]. Furthermore, chitin combined to graphene oxide as aerogel beads effectively absorbed excessive bilirubin in the blood [60]. Chitin nanogel with rectorite nanocomposite stopped bleeding within 121 s in rat tail vein, and promoted higher hemostatic activity compared to chitosan-based hemostatic products [61]. Furthermore, Chitin derived from demosponge *Ianthella labyrinthus* and chitin derived from spider *Caribena versicolor* were effectively served as a 3D scaffold for culturing induced pluripotent stem-cell-derived cardiomyocytes as well as commercial extracellular matrix [62,63].

It has been known that orally intake of 5% chitosan in the diet reduces serum cholesterol and atherogenesis inhibition in apolipoprotein E-deficient mouse model [64]. Chitosan as a supplemental diet downregulated the markers involving obesity such as leptin in high fat diet rats [65]. Chitosan oligosaccharide decreased serum cholesterol by promoting accumulation of cholesterol in liver, bile, and feces with reverse cholesterol transport pathway [66]. It is known that chitosan oligosaccharide reduces serum cholesterol. Chitosan oligosaccharide increased cell surface expression of low-density lipoprotein receptor (LDLR) and increased lipid droplets in HepG2 cells, suggesting that chitosan oligosaccharide effectively reduced serum lipids by facilitating the accumulation of lipids into the cells [67]. Furthermore, chitosan oligosaccharide downregulated mRNA expression of LPS-induced E-selectin and intercellular adhesion molecule-1, which are a part of the inflammatory responses in endothelial cells via MAPK inhibition [68]. Oral intake of chitosan oligosaccharide also reduced the marker of atherosclerosis including the lesion area in aorta or plaque area in aortic roots, and greatly reduced cholesterol and triglyceride in apolipoprotein E deficient mice (apoE−/−) [69]. The combination of chitosan oligosaccharide ingestion and exercise such as running improved immune system by increasing spleen to body weight ratio and lung to body weight ratio compared to water gavage only in Sprague-Dawley (SD) rats [70]. Interestingly, chitosan improved blood perfusion and promoted neovascularization by modulation of gut microbiota in a mouse hindlimb ischemia model [71]. Furthermore, chitosan was used as a vehicle for drug delivery for transportation of doxorubicin to improve the treatment of blood malignancies [72]. Chitosan in the form of synthetic CD47 antibody-chitosan/hyaluronic acid polyelectrolyte complex inhibited atherosclerotic plaques with downregulated NLRP3 inflammasome expression in apolipoprotein E deficient mice (apoE−/−) [73].

### 2.3. Respiratory Diseases

It is well known that penetration of chitin into human bodies causes the production of chitinase, the enzyme that modulates immune response, and chitinase-like proteins YKL-40. YKL-40 was associated with increased severity in asthmatic patients [74]. YKL-40 level was also positively correlated with neutrophils, sputum IL-1β, and plasma IL-6 [75]. Moreover, chitin from fungi induced eosinophilic infiltration in a mouse model [76]. Chitin also promoted proinflammatory response by inducing proinflammatory cytokine release including IL-25 and IL-33 in human bronchial epithelial cells [77]. Chitin has been known as an adjuvant for immune response. It was demonstrated that chitin from house dust mite promoted airway hypersensitivity in ovalbumin-induced airway inflammation via a TLR-2 dependent pathway [78]. Furthermore, it is known that chitin is the major component of fungi in their cell wall. Chitin exposure induced macrophage activation which upregulates the expression of chitin degrading enzyme chitotriosidase [79]. Chitotriosidase was also involved in lung diseases such as tuberculosis, chronic obstructive lung diseases [80]. Apart from chitotriosidase, acute exposure to the fungal pathogen *Aspergillus fumigatus* promoted the function of acidic mammalian chitinase, which determined the severity of fungal asthma [81]. Therefore, inhibition of chitotriosidase and acidic mammalian chitinase is regarded as a drug target for respiratory diseases [82]. However, it has been shown that a chitin analog AVR-25 partially alleviated pulmonary dysregulation in a hyperoxia-induced experimental mouse model of bronchopulmonary dysplasia by suppressing inflammation [83]. These findings indicate that chitin may have both beneficial and detrimental effects.

In contrast to chitin, chitosan oligosaccharides have been demonstrated to have potential beneficial effects on respiratory diseases. Oral intake of chitosan oligosaccharide (500 mg/kg) at a single dose alleviated particulate matter (PM) 2.5-induced lung inflammation by decreasing lactate dehydrogenase, IL-8, and TNF-α in PM 2.5-induced rat model [84]. Chitosan oligosaccharide (100 kDa and 90% deacetylation) prevented inflammation, oxidative stress and apoptosis in the lung tissues of blast injury-induced mice by diminishing protein expression of p38 and ADMA (an inhibitor of endogenous nitric oxide synthase that positively correlated with hypertension) and recovering dimethylarginine dimethylaminohydrolase 1 (DDAH1), a hydrolase of ADMA [85]. Furthermore, oral intake of 16 mg/kg/day of low molecular weight chitosan oligosaccharide reduced IgE-induced airway inflammation in mice by downregulating both protein level and mRNA level of proinflammatory cytokines including IL-4, IL-5, IL-13, and TNF-α [86].

### 2.4. Renal Diseases

There are reports demonstrating the effect of chitin on renal diseases. Oral ingestion of chitin as surface-deacetylated chitin nano-fiber (40 mg/kg/day) reduced uremic toxins including oxidants in nephrectomized rats [87]. However, upregulated YKL-40 level in urine and plasma represents the biomarker of acute kidney injury [88,89]. In contrast, chitosan and chitosan oligosaccharide have potential therapeutic effects on kidney diseases. The combination of chitosan with gynostemma and motherwort was used for protecting chronic renal failure by inhibiting inflammation in adenine-induced rat chronic renal failure [90]. Chitosan incorporated with gallic acid reduced the formation of calcium oxalate crystal, which was mainly a kidney stone, and had antioxidative effects [91]. Chitosan as a cat diet improved kidney function and quality of life in elderly cats with 3 and 4 International Renal Interest Society (IRIS) stages [92]. It was also shown that chitosan oligosaccharide improved kidney function in streptozotocin-induced diabetic rats [93]. It was found that 0.1% chitosan oligosaccharide with more than 90% deacetylation prevented glycerol-induced acute renal failure in rats by decreasing renal dipeptidase activity, a diagnostic marker of acute renal failure [94]. Furthermore, chitosan oligosaccharide with carboxymethyl group relieved renal injury induced by doxorubicin and promoted antioxidative effects in rats [95]. Interestingly, chitosan oligosaccharide triggered G2/M phase arrest, promoted endoplasmic reticulum stress pathway, and inhibited tumor growth in human renal carcinoma cells and xenograft tumor models [96]. Moreover, chitosan oligosaccharide had a chelating property which detoxified depleted uranium cytotoxicity in human renal proximal tubular epithelial cells [97]. Recently, chitosan oligosaccharide at the concentration of 50 and 100 µg/mL was shown to reduce renal cyst growth via CaMKKβ-induced AMPK activation without cytotoxicity [98]. Furthermore, the detoxification property of chitosan has been applied in hemodialysis patients. The ingestion of chitosan decreased the level of indoxyl sulfate and phosphate by binding to these molecules [99]. Chitosan has also been applied as siRNA delivery system targeting kidney. For instance, chitosan nanoplex effectively covered siRNA for knocking down PDGF-B and PDGFR-beta [100]. Chitosan was also used as biomaterials in combination with collagen for culturing human renal proximal tubular cells [101]. The potential beneficial effects of chitosan oligosaccharide on kidney diseases have been also reviewed elsewhere [102]. 

### 2.5. Gastrointestinal Diseases and Gut Microbiota

Many previous studies demonstrated the potential effects of chitin and chitosan oligosaccharide on gastrointestinal disease. It was shown that chitin protected intestinal barrier function in DSS-induced colitis in a protochordate model [103]. Chitin combined with glucan (chitin-glucan complex) was used as a prebiotic. This chitin-glucan complex improved the growth of *Bifidobacterium*, a probiotic, in a rat model [104]. Interestingly, intake of 4.5 g/day of chitin-glucan in food for 3 weeks increased beneficial microbiota metabolites including butyric, iso-valeric and caproic acids without major changes in gut microbiota composition [105]. Chitin has also been applied as surface-deacetylated chitin nanofiber. It was shown that oral intake of 80 mg/kg/day of surface-deacetylated chitin nanofiber decreased hepatic injury and oxidative stress in a nonalcoholic steatohepatitis model of rats [106]. 

Chitosan and chitosan oligosaccharide have beneficial effects on gastrointestinal tract, especially as nutritional supplements for animals and food supplements for human. In livestock, chitosan oligosaccharide has been used as feed additives in animal diet. It was shown that 100 mg/kg of dietary chitosan oligosaccharide supplementation promoted growth performance, reduction of diarrhea, nutrient digestibility and attenuation of *E.coli* K88 infection in weaning pigs [107,108]. The combination of chitosan and zinc at the dose of 100 mg/kg as a feed additive in diet also promoted the activities of digestive enzymes such as amylase, reduced diarrhea and improved growth performance in weaning pigs [109]. However, chitosan mixed with probiotic such as *Enterococcus faecalis* did not significantly reduce severity of diarrhea and affect growth performance in *E.coli* K88-inoculated weaning pigs [110]. Moreover, high molecular weight chitosan oligosaccharide (20 to 30 kDa) just only increased ZO-1 expression and decreased the mRNA expression of IL-1β and TNFα in weaning pigs without affecting diarrhea, average dairy gain, gain to feed ratio, and antioxidant capacity [111], suggesting that specific forms of chitosan oligosaccharide gives the therapeutic effects in weaning pig. Furthermore, low molecular weight chitosan oligosaccharide (8 kDa) with 90% deacetylation improved gut absorption, increased villus length and promoted intestinal cell proliferation in weaning pig [112]. Oral intake of 200 mg/kg/day of chitosan oligosaccharide in drinking water also protected gut from the modulation of glucose metabolism and gut dysbiosis in diabetic mice [113]. At a cellular level, 100 µg/mL of low molecular wight chitosan oligosaccharide (approximately 5 kDa) accelerated tight junction assembly and inhibited cholera toxin-induced intestinal fluid secretion via CaSR-PLC-IP3 receptor channel-mediated AMPK activation in intestinal epithelial cells [114]. Chitosan oligosaccharide suppressed mRNA expression of proinflammatory cytokines, and inhibited the downregulation of PPARγ in palmitic acid-induced HepG2 cells and high fat diet mice [115]. Many reports reveal that chitosan and chitosan oligosaccharide modulate gut microbiota. Chitosan prevented gut dysbiosis and inhibited the activation of toll-like receptor and nod-like receptor signaling pathway in high fat diet rats [116]. However, molecular weight and degree of deacetylation of chitosan oligosaccharide are major determinants of effects on human gut microbiota composition as well as therapeutic effects. For instance, a highly deacetylated chitosan oligosaccharide decreased *Bifidobacterium* spp., *E. rectale/C. coccoides, C. histolyticum* and *Bacteroides/Prevotella* populations in human gut [117]. Chitosan oligosaccharide with >95% deacetylation reduced *Lactobacillus, Bifidobacterium* and *Desulfovibrio*, deleterious bacteria that were correlated with inflammatory bowel disease [118], and increased abundance of *Akkermansia* that was a good bacteria [119]. The 3 kDa chitosan oligosaccharide diminished gut dysbiosis and downregulated mRNA expression of proinflammatory cytokines in azoxymethane and dextran sulfate sodium-induced mouse model of colorectal cancer [120]. Moreover, chitosan oligosaccharide ameliorated hepatic steatosis and liver injury, and reduced triglyceride and free fatty acid in diet-induced obese mice by downregulating inflammatory genes and modulation of gut microbiota [121]. Furthermore, chitosan protected liver from ischemia-reperfusion injury via regulating Bcl-2/Bax, TNF-α and TGF-β expression [122], prevented lipid metabolic disorder by combination with Ganoderma polysaccharide [123], alleviated menopausal symptoms [124], and protected the gut from ischemic symptoms [71]. High molecular weight chitosan was also prepared as nanoparticles for drug delivery in gut. For instance, 400 kDa chitosan integrated to insulin loaded chitosan nanoparticles prolonged bioavailability of insulin release [125]. Interestingly, chitosan nanoparticle was also used as a food supplement for improvement of growth performance and immunity in weaning pigs [126].

Apart from chitin, chitosan, and chitosan oligosaccharide, there are other polysaccharides that have therapeutic effects on gut. For instance, mannan oligosaccharide (10 µM) promoted intestinal barrier function in T84 cells via AMPK activation [127,128]. Fructo-oligosaccharide (0.1 mg/mL), a prebiotic, accelerated intestinal tight junction reassembly via AMPK activation in T84 cells [129]. 

### 2.6. Endocrinological Diseases and Diabetes Mellitus

Chitin combined with other compounds has been shown to possess therapeutic effects. For instance, oral intake of 4.5 g/day of chitin combined with glucan reduced oxidized low-density lipoprotein in human subjects [130]. Furthermore, chitin has been applied as a biomaterial for prolonging bioavailability. For instance, injectable thermo-sensitive hydrogel based on hydroxypropyl chitin was incorporated to salmon calcitonin to extend long-term sustained salmon calcitonin release [131].

Likewise, chitosan has been combined with other compounds that possess antidiabetic effects. For instance, 3-*O*-sulfochitosan was reported to reduce blood glucose in diabetic rats [132]. Chitosan combined with metformin, a type 2 diabetic drug, synergistically enhanced drug efficacy and reduced lethal effects of drug overdose [133]. Furthermore, chitosan oligosaccharide has been applied for drug delivery system in diabetes. For instance, chitosan-microcapsulated insulin, chitosan-stabilized selenium nanoparticles, chitosan encapsulated resveratrol, chitosan coating of TiO_2_ nanotube arrays for metformin, chitosan nanoparticle and chitosan hydrogel were used for improving diabetic therapy [134,135,136,137,138,139,140,141,142]. Interestingly, the development of chitosan oligosaccharide as a drug or supplement for diabetic treatment has been studied for a decade. The proteomic data demonstrated the antidiabetic effects and anti-obesity of orally intake chitosan oligosaccharide in ob/ob mice [143]. Oral intake of chitosan oligosaccharide (>90% deacetylation; 500 mg/kg) promoted insulin sensitivity in streptozotocin-induced diabetic rats. Chitosan oligosaccharide (100 mg/L) also promoted cell proliferation in primary culture islet cells and pancreatic β-cell lines [144]. Moreover, the low molecular weight chitosan oligosaccharide (~1.2 kDa) promoted cell proliferation in primary culture islet cells and pancreatic β-cell lines as well as improving insulin sensitivity greater than the high molecular weight chitosan oligosaccharide [145]. Moreover, chitosan oligosaccharide with a molecular weight of 1.3 kDa and 55% deacetylation was used as an oral insulin delivery system that showed the highest effect of glucose reduction [146]. Furthermore, chitosan oligosaccharide has been used as a food supplement for diabetic treatment. For instance, GO2KA1, a commercial chitosan oligosaccharide supplement, had a beneficial effect on glucose control in subjects with prediabetes by regulating postprandial glucose [147]. GO2KA1 promoted glucose uptake into intestinal epithelial cells, enhanced adipocyte differentiation, and upregulated PPARγ expression [148]. GO2KA1 has been used in clinical trials. It was found that GO2KA1 effectively reduced postprandial blood glucose levels in subjects with impaired glucose tolerance and impaired fasting glucose [149]. However, it remains unclear whether chitosan oligosaccharide has a direct or indirect antidiabetic effect. It was also shown that chitosan oligosaccharide exerted antidiabetic effects via gut microbiota modulation [116]. Chitosan oligosaccharide was used as a supplementary drug for improving the glycemic control of sitagliptin in type 2 diabetes mellitus (T2DM) by reducing insulin resistance and proinflammatory cytokines, and increasing insulin sensitivity [150]. Chitosan oligosaccharide combined with xanthine derivatives improved liver and kidney functions compared to pioglitazone, a standard antidiabetic drug [151]. Chitosan oligosaccharide also upregulated the expression of browning genes in white adipose tissues and thermogenesis of brown adipose tissues, which consequently reduced obesity in obese rats [152]. Chitosan oligosaccharide was applied in the form of tablet that had therapeutic effects on the regulation of serum lipid level and downregulation of cholesterol excretion genes including CYP7A1, LXR, PPAR-α, and LDLR in high fat diet-induced hyperlipidemic rats [153]. Moreover, chitosan oligosaccharide reduced endoplasmic reticulum stress in HepG2 cell lines [154]. Interestingly, chitosan oligosaccharide did not induce any hepatotoxic effects or lipid metabolism disorders in normal Sprague-Dawley rats [155]. 

### 2.7. Inflammatory Diseases

Inflammation is generally a crucial defense mechanism of human body against pathogens, injuries, or toxins. In addition, there are several diseases related to hyperinflammatory responses such as inflammatory bowel disease and systemic lupus erythematosus. Chitin has been applied as a biomaterial for suppressing inflammation. For instance, chitin nanofibril was used for inhibiting skin inflammation in the experimental atopic dermatitis mouse model by suppression of NF-κB [156]. However, many reports have demonstrated that chitin is an inflammatory inducer. For instance, chitin induced inflammation in peripheral blood mononuclear cells from obese subjects ex vivo [157]. Chitin triggered inflammatory responses via type 2 innate lymphoid cells and γδ T cell activation [158]. Furthermore, chitin enhanced IL-33 secretion and consequently IL-1β secretion by dendritic cells in ovalbumin-induced asthmatic mice [159]. However, Wagener el al demonstrated that fungal chitin triggered anti-inflammatory cytokines including IL-10 via NOD2 and TLR-9 activation, indicating that chitin exposure triggered inflammatory responses together with anti-inflammatory responses as negative feedback to regulate the inflammatory process. Interestingly, low size chitin (1-10 µm) induced secretion of IL-10, an anti-inflammatory cytokine, at low concentrations, but induced secretion of TNFα, a proinflammatory cytokine, at high concentrations [160]. However, chitin nanofibrils, nanorods structure of chitin, downregulated proinflammatory cytokines including TNF-α, IL-1α, IL-1β, IL-6, and IL-8, and concomitantly upregulated antimicrobial peptide β-defensin 2 in human keratinocytes (HaCaT cells) [161].

Chitosan and chitosan oligosaccharide have been reported as anti-inflammatory agents. Chitosan recovered intestinal barrier function in DSS-induced colitis by stimulating expression of tight junction proteins such as claudin-1, occludin, and ZO-1 [162]. Chitosan downregulated chitinase enzyme YKL-40 in primary human macrophages [163]. Carboxymethyl chitosan was shown to have anti-inflammatory effects in mice [164]. Likewise, it was shown that an oral intake of chitosan oligosaccharide (20 mg/kg/day) alleviated DDS-induced acute and chronic colitis in mice by inhibiting an NF-κB pathway [165]. Moreover, chitosan oligosaccharide downregulated NF-κB downstream targets such as COX-2 and upstream targets such as TLR-4 in lipopolysaccharide-induced inflammation in intestinal epithelial cells [166]. Furthermore, 50-200 µg/mL of a highly *N*-acetylated chitosan oligosaccharide inhibited protein expression of PI3K/Akt signaling pathway, which was involved in proinflammatory cytokine production in RAW 264.7 macrophage cells [167]. The physiochemical properties as well as preparation processes of chitosan oligosaccharide influence its anti-inflammatory effect. It was shown that 42% fully deacetylated oligomers plus 54% monoacetylated oligomers of chitosan oligosaccharide alleviated inflammation, whereas 50% fully deacetylated oligomers plus 27% monoacetylated oligomers promoted inflammation in RAW 264.7 macrophage cells [168]. Furthermore, chitosan oligosaccharide protected against shrimp tropomyosin-induced food allergy by downregulation of IL-4, IL-5, and IL-13 and upregulation of IFN-γ in sensitized mice [169]. Chitosan oligosaccharide (200 mg/kg) prevented heat stress-induced inflammatory responses by decreasing liver IL-1β concentration [170]. Apart from chitin and chitosan oligosaccharide, fructooligosaccharide and yeast polysaccharide had an inhibitory effects on TNF-α-induced GLP-1 secretion in L cells and DSS-induced colitis in mice, respectively [171,172].

### 2.8. Cancer

Chitin has promise for development as an anti-cancer agent and a vehicle for anticancer drug delivery. It was shown that chitinase-3 like protein-1 (CHI3L1), which was upregulated and promoted proinflammatory mediators in breast cancer cells, was inhibited by chitin [173,174]. Chitin downregulated vascular endothelial growth factor C (VEGF-C) synthesis that was related to tumor angiogenesis [175]. In addition, chitin has been prepared in various forms that can counteract cancer. For instance, silver embedded chitin nanocomposites promoted cytotoxicity in human breast cancer (MCF-7) cells [176]. Chitin-glucan-aldehyde-quercetin conjugation induced cytotoxicity in a macrophage cancer cell line (J774) with no toxic effect on peripheral blood mononuclear cells (PBMCs) [177]. Furthermore, chitin has been used for anticancer drug delivery. For instance, chitin with poly L lactic acid composite nanogel containing doxorubicin induced cytotoxicity in liver cancer HepG2 cells and enhanced anticancer drug efficacy [178]. Chitin nanoparticles were loaded with anticancer natural product ellagic acid, which inhibited breast cancer cell growth [179]. 

Interestingly, there are several reports demonstrating the potential antitumor effect of chitosan and chitosan oligosaccharide. For instance, chitosan decreased cell proliferation, stimulated apoptotic effects, and decreased cell adhesion in human melanoma cell lines including SKMEL38 cells, RPMI7951 cells, and A375 cells, respectively [180]. Oral intake of 500 mg/kg/day of chitosan oligosaccharide abolished tumor progression in colitis-associated colorectal cancer via NF-κB inhibition and AMPK activation [181]. Chitosan oligosaccharide modulated cell autophagy that inhibited cell proliferation of A549 lung cancer cell line [182]. Low molecular weight chitosan oligosaccharide induced cytotoxic effects, cell cycle arrest and apoptosis in oral squamous cell carcinoma (SCC) cells without any effects on noncancerous keratinocyte (HaCaT) cell lines [183]. Chitosan also had anticancer activity in various types of cancer such as human ovarian cancer, breast cancer and cervical carcinoma [184,185,186]. Chitosan was combined with other compounds to enhance anticancer effects. For instance, carboxymethyl chitosan inhibited tumor growth in mouse hepatocarcinoma by abolishing tumor angiogenesis [187]. Chitosan selenate inhibited cancer cell viability and promoted cancer cell apoptosis in lung cancer A549 cells [188]. Furthermore, 5-fluorouracil-conjugated chitosan oligosaccharide plus vanillin, indomethacin-conjugated chitosan oligosaccharide nanoparticles, and thioguanine-conjugated chitosan graphene oxide were applied for cancer drug delivery systems [189,190,191]. 

### 2.9. Aging

The world population is beginning to age. Antiaging agents have been developed to support the aging society. Oxidative stress is a major promoting factor of aging. Antioxidant compounds are recognized as therapeutic agents for delay aging. Chitin had a scavenging activity to chelate 1,1-diphenyl-2-picrylhydrazyl radicals [192]. Chitin was also used as a biomaterial for antioxidant agent container. For instance, chitin nano-crystal complex containing melatonin, vitamin E, and β-glucan reduced wrinkle and yielded a better skin appearance in human subjects [193]. Chitin-glucan-aldehyde-quercetin conjugates also had a potent antioxidant activity [177]. Interestingly, chitin nanofibrils and nanochitin can mimic the extracellular matrix, so these agents have been applied as cosmeceuticals against aging [194]. 

Chitosan oligosaccharide has widely been used as antioxidative agents. Chitosan supplementation reduced oxidative stress in the heart tissues and maintained glutathione reductase, glutathione peroxidase, and reduced glutathione in young and aged rats [195]. Chitosan oligosaccharide restored redox balance in LPS-induced oxidative stress [196]. Chitosan oligosaccharide with 90% deacetylation potently inhibited oxidative stress in rats [197]. Several studies confirmed the therapeutic effects of chitosan and chitosan oligosaccharide on oxidative stress in many types of animal models including aging mice, weaning pig, hydrogen peroxide-induced rats, and heat-stressed rats [198,199,200,201]. Interestingly, chitosan oligosaccharide recovered aging-induced liver dysfunction via the upregulation of Nrf2 antioxidant signaling [202]. Chitosan-gallic acid, synthetic carboxymethyl chitosan, chitosan-ellagic acid and selenide chitosan sulfate have also been demonstrated to have an antioxidant activity [203,204,205,206]. 

### 2.10. Infectious Disease

Generally, chitin has no antipathogenic effects [207]. Although, chitin derived from demosponges *Ianthella flabelliformis* was applied as drug delivery material for antibiotic drug including decamethoxine [208]. Additionally, many reports have demonstrated the antibacterial effects and antifungal effects of chitosan oligosaccharide. Chitosan oligosaccharide (10 kDa) had the highest antimicrobial effect with minimum inhibitory concentration values of 32–64 μg/mL on *Propionibacterium acnes* [24]. In addition, low-molecular-weight chitosan oligosaccharide inhibited bacterial activity, biofilm formation and hemolytic activity of *Staphylococcus aureus* [209]. In vivo and in vitro models showed that chitosan reduced *Cryptosporidium parvum* oocyst viability and *Cryptosporidium parvum* multiplication, respectively [210,211]. Furthermore, chitosan oligosaccharide killed *Candida auris* in both nonaggregative form (NCPF 8973) and aggregative form (NCPF 8978) [212]. Interestingly, chitosan oligosaccharide with an averaged degree of polymerization of 32 and a fraction of acetylation of 0.15 inhibited *Candida* spp. growth [213]. Interestingly, chitosan oligosaccharide had antifungal effects against *Ceratocystis fimbriata* by promoting fungal apoptotic cascades including ROS accumulation, mitochondrial dysfunction, and caspase activation [214]. Furthermore, *N,N,N*-trimethyl-*O*-(ureidopyridinium) acetyl chitosan derivatives, chitosan oligosaccharide functionalized silver nanoparticles, chitosan oligosaccharide-capped gold nanoparticles, and chitosan oligosaccharide-*N*-chlorokojic acid mannich base polymer were shown to hold promise for antibacterial application [215,216,217,218]. 

### 2.11. Trauma/Wound

Chitin has been applied as a major component of biomaterials for wound healing. Chitin was used for suturing because of its safety and rapid tissue recovery [219]. Chitin derived from demosponges *Ianthella labyrinthus* and *Aplysina archeri* was applied as alternative gauze fabrics, and it effectively absorbed blood into chitinous microtubes [62,220]. Additionally, several studies improved efficacy of chitin for wound healing by integration with other components. For instance, diacetyl chitin, a novel absorbable surgical suture, was ultimately absorbed within 42 days after suturing without tissue reaction, and promoted faster skin regeneration in vivo [221]. Chitin hydrogel, acrylamide-modified-β-chitin with alginate dialdehyde, showed promising properties including biocompatibility, biodegradability and injectability, and effectively accelerated wound healing [222]. In addition, sacchachitin nanofibers accelerated blood clotting times by 30 s and significantly promoted wound healing in streptozotocin-induced diabetic rats [223]. Interestingly, chitin accelerated wound healing via a MyD88-dependent pathway, followed by a TGF-β/Smad pathway [224]. Furthermore, *Pseudomonas aeruginosa*-infected wounds in db/db diabetic mice were diminished by cleaning with cleansing agent hypochlorous acid and covering with silver nanoparticle/chitin in the form of nanofiber sheet [225]. Chitin-amphiphilic ion/quaternary ammonium salt having antibacterial and antipollution effects was also used for wound healing [226]. Chitin-lignin gels, as an extracellular matrix-like scaffold, were applied as wound dressing material that had a property of sustainable drug release especially antibiotics [227]. Recently, chitin has been applied as a tissue adhesive. Chitin nanowhiskers with a Schiff base crosslinking hydrogel of carboxymethyl chitosan and dextran dialdehyde enhanced tissue adhesive strength with no cytotoxicity and with antibacterial effects [228]. Furthermore, chitin nanofibrils have been also applied as biomaterials. For instance, addition of 0.5% chitin nanofibrils into chitosan sponges promoted the stopping of arterial bleeding faster than commercial hemostatic agents [229].

Likewise, chitosan and chitosan oligosaccharide have been demonstrated as a biomaterial for promoting wound healing. For instance, chitosan oligosaccharide incorporated with silver nanoparticles accelerated wound healing by activating TGF-β/Smad pathway [230,231]. Chitosan was further used as a nanoparticle for carrying drugs including pioglitazone, heparin and bemiparin for wound healing application especially for diabetic wounds [232,233]. Chitosan-polyurethane hydrogel membrane in combination with mononuclear bone marrow fraction cells was used for wound healing in a diabetic rat model [234]. Furthermore, chitosan-curcumin complex, quaternary ammonium chitosan nanoparticles, carboxymethyl chitosan plus alginate were developed as biomaterials with high potential for wound healing application [235,236,237]. 

## 3. Limitation

The limitation in utilization of chitin is its potential toxicity in the human body, especially in the respiratory tract. According to previous studies, chitin exposure in the respiratory tract induced chitinase production, which was positively correlated to asthma [76]. In addition, chitin exposure to the respiratory tract can trigger innate immune response, especially macrophage and eosinophil activation. Furthermore, chitin is insoluble in water. However, oral intake of 5% chitin in a diet for 13 weeks showed no apparent toxicity in the gastrointestinal tract in rats [238]. Therefore, it appears that the adverse effects of chitin depend on the route of exposure. 

In contrast to chitin, several studies have reported that oral ingestion of chitosan and chitosan oligosaccharide shown minimal toxicity [239,240]. Furthermore, chitosan oligosaccharide can dissolve in water. Chitosan and chitosan oligosaccharide have been approved as food additives by the American Food and Drug Administration (FDA). The potential systemic toxic effects of chitosan oligosaccharide are minimal.

## 4. Conclusions and Future Perspectives

**Figure 1:**
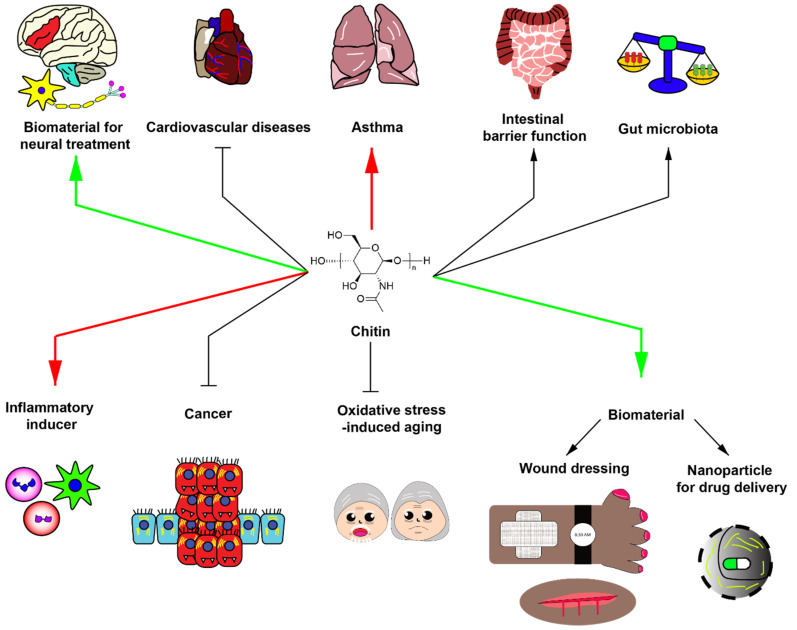
Potential roles of chitin in biomedical applications. Green arrows indicate potential beneficial applications of chitin with strong evidence. Red arrows demonstrate potential detrimental effect of chitin with strong evidence. Black arrows delineate the potential effects of chitin with slight evidence. Chitin has been generally utilized as biomaterials for neural treatment, wound dressing and nanoparticle component for drug delivery. In addition, chitin has been used for improving intestinal barrier function, increasing beneficial gut microbiota, partially inhibiting cardiovascular diseases, inhibiting cancer growth, and inhibiting oxidative stress-induced aging. However, chitin may have detrimental effects. For instance, chitin exposure can induce asthma and acts as an inflammatory inducer.

Currently, chitin and chitosan have been used as a biomaterial for wound healing and drug delivery. Moreover, chitin and chitosan have been integrated with other chemicals to improve efficacy in therapeutic applications. Interestingly, the thermostability of chitin, chemical tolerance of chitin, and accessible natural source of chitin shed light on various procedures for biomaterial generation. Based on these advantageous properties, further development of chitin is needed to provide better biomaterials applicable for various human diseases. However, the development of chitin for medical applications requires further steps to improve safety and efficacy. A summary of potential applications and adverse effects of chitin is shown in Figure 1. 

**Figure 2:**
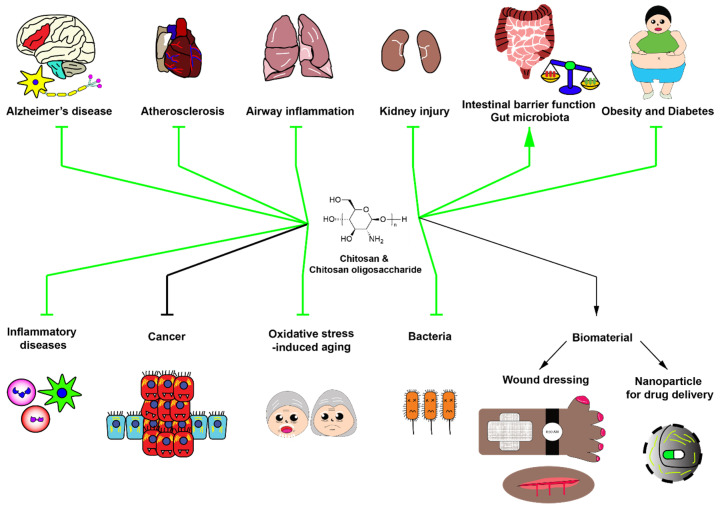
Potential roles of chitosan and chitosan oligosaccharide in biomedical applications. Green arrows indicate the beneficial applications of chitosan and chitosan oligosaccharide with strong evidence. Black arrows indicate the potential effects of chitosan and chitosan oligosaccharide with slight evidence. Chitosan and chitosan oligosaccharide have numerous beneficial effects including anti-Alzheimer’s disease, anti-atherosclerosis, anti-inflammatory diseases, kidney injury alleviation, improvement of intestinal barrier function, enhancement of beneficial gut microbiota, alleviation of obesity and type II diabetes, prevention of inflammatory diseases, anticancer activity, antiaging activity and antibacterial activity, and serve as valuable biomaterials for wound dressing and drug delivery.

Chitosan oligosaccharide has been applied for treatment of several types of human diseases or pathological conditions. Chitosan oligosaccharide with low molecular weight (<5 kDa) and >90% degree of deacetylation inhibits inflammatory responses by promoting anti-inflammatory pathways. Furthermore, chitosan oligosaccharide has antioxidative and anticancer effects. Interestingly, chitosan oligosaccharide, as a prebiotic for gut microbiota, modulates gut microbiota leading to the alleviation of systemic diseases, especially atherosclerosis. Biological effects of these compounds are determined by degree of deacetylation and polymerization, which remains the challenge in the development of chitosan oligosaccharide as an effective food supplement. Further investigations are needed to reveal detailed molecular/cellular mechanisms of biological effects of these polymers especially the role of their prebiotic effect and their direct effect on disease-specific cells or drug targets. Finally, since chitosan oligosaccharide possesses antioxidative effects, its potential application as an anti-aging agent should be further investigated. Potential applications and adverse effects of chitosan and chitosan oligosaccharide are summarized in Figure 2.
